# Targeting a phospho-STAT3-miRNAs pathway improves vesicular hepatic steatosis in an *in vitro* and *in vivo* model

**DOI:** 10.1038/s41598-018-31835-2

**Published:** 2018-09-11

**Authors:** Laura Belloni, Silvia Di Cocco, Francesca Guerrieri, Abigail D. G. Nunn, Silvia Piconese, Debora Salerno, Barbara Testoni, Claudio Pulito, Federica Mori, Matteo Pallocca, Andrea Sacconi, Elisa Vivoli, Fabio Marra, Sabrina Strano, Giovanni Blandino, Massimo Levrero, Natalia Pediconi

**Affiliations:** 1 Center for Life Nano Science@Sapienza, Istituto Italiano di Tecnologia, Rome, Italy; 2 Department of Internal Medicine - DMISM, Sapienza University, Rome, Italy; 3 Department of Molecular Medicine, Sapienza University, Rome, Italy; 4 Pasteur Institute Italy-Fondazione Cenci Bolognetti, Rome, Italy; 5 Cancer Research Center of Lyon (CRCL), UMR INSERM U1052 - CNRS 5286, Lyon, France; 6 Molecular Chemoprevention Group, National Cancer Institute Regina Elena, Rome, Italy; 7 UOSD SAFU, National Cancer Institute Regina Elena, Rome, Italy; 8 Oncogenomic and Epigenetic Unit, National Cancer Institute Regina Elena, Rome, Italy; 9 Department of Clinical and Experimental Medicine, University of Florence, Florence, Italy; 10 Department of Oncology, Juravinski Cancer Center-McMaster University, Hamilton, ON Canada; 11 Hepato-Gastroenterologie, Hopital de la Croix-Rousse, Hospices Civils de Lyon, Lyon, 69004 France

## Abstract

Non-alcoholic fatty liver disease (NAFLD) is a leading cause of chronic liver disease. Although genetic predisposition and epigenetic factors contribute to the development of NAFLD, our understanding of the molecular mechanism involved in the pathogenesis of the disease is still emerging. Here we investigated a possible role of a microRNAs-STAT3 pathway in the induction of hepatic steatosis. Differentiated HepaRG cells treated with the fatty acid sodium oleate (fatty dHepaRG) recapitulated features of liver vesicular steatosis and activated a cell-autonomous *inflammatory response*, inducing STAT3-Tyrosine-phosphorylation. With a genome-wide approach (Chromatin Immunoprecipitation Sequencing), many phospho-STAT3 binding sites were identified in fatty dHepaRG cells and several STAT3 and/or NAFLD-regulated microRNAs showed increased expression levels, including miR-21. Innovative CARS (Coherent Anti-Stokes Raman Scattering) microscopy revealed that chemical inhibition of STAT3 activity decreased lipid accumulation and deregulated STAT3-responsive microRNAs, including miR-21, in lipid overloaded dHepaRG cells. We were able to show *in vivo* that reducing phospho-STAT3-miR-21 levels in C57/BL6 mice liver, by long-term treatment with metformin, protected mice from aging-dependent hepatic vesicular steatosis. Our results identified a microRNAs-phosphoSTAT3 pathway involved in the development of hepatic steatosis, which may represent a molecular marker for both diagnosis and therapeutic targeting.

## Introduction

Non-alcoholic fatty liver disease (NAFLD) is the most common chronic liver disease in developed countries^1^. The excessive accumulation of triglyceride-containing lipid droplets (LD) within hepatocytes in NAFLD patients is a potentially reversible process, that may evolve into a severe necro-inflammatory form called non-alcoholic steatohepatitis (NASH), which can eventually lead to cirrhosis and hepatocarcinoma (HCC)^2,3^. Genetic and epigenetic factors together with multiple risk factors (i.e. obesity, insulin resistance, type-2-diabetes, elevated glucose, hyperlipidemia) have been described to promote liver injury^1,4^. Intrahepatic activation of pro-inflammatory signaling, such as the IL6/STAT3 pathway, plays a significant role in the development of chronic liver diseases. STAT3 is constitutively activated in HCC by phosphorylation of Tyr705 and Ser727, resulting in STAT3 dimerization, nuclear translocation, DNA binding and gene transcription^5,6^. IL6/STAT3-dependent molecular mechanisms driving hepatic steatosis and the pathological processes that cause NAFLD progression are still poorly understood^3,7^.

MicroRNAs (miRNAs) are a class of noncoding endogenous RNAs that regulate gene expression and specific miRNA expression profiles are strongly associated with several pathological conditions, including NAFLD^8,9^. There is a growing body of evidence demonstrating that miRNAs are closely associated with the IL6/STAT3 signaling pathway, supporting the existence of regulatory feedback loops between expression of specific miRNAs and the STAT3 pathway in HCC and other diseases^10,11^.

The present study investigates the role of a miRNAs-phospho-STAT3 pathway in the induction of liver vesicular steatosis in an *in vitro* model, represented by hepatic differentiated HepaRG cells treated with sodium oleate, fatty dHepaRG. Indeed, lipid overloaded dHepaRG cells showed typical features of mature hepatocytes, as we observed by CARS (Coherent Anti-stokes Raman Scattering) microscopy, an innovative technique that enables lipid droplets visualization and quantification without labeling, by probing their characteristic vibrational properties^12^.

We identified by Chromatin Immunoprecipitation Sequencing that phospho-STAT3, which is activated in fatty dHepaRG, is able to bind and regulate several miRNAs after lipid overload. Our results showed for the first time that suppression of the miRNAs-phospho-STAT3 pathway by S3I-201, a STAT3 specific inhibitor, decreased hepatic lipid accumulation. We have also observed *in vivo* that reducing both phospho-STAT3 and miR-21 levels in C57BL6 mice liver, by long-term treatment with metformin, a widely used anti-diabetic drug, strongly improved age dependent hepatic steatosis. Activation of a new miRNAs-phospho-STAT3 pathway that is involved in the development of hepatic steatosis may represent a target for novel therapeutic strategies and might also have potential as a biomarker for diagnosis.

## Results

### Sodium oleate treatment of dHepaRG cells induced lipid droplets accumulation, ROS generation and deregulated lipid metabolism and liver-specific genes expression

**Figure 1:**
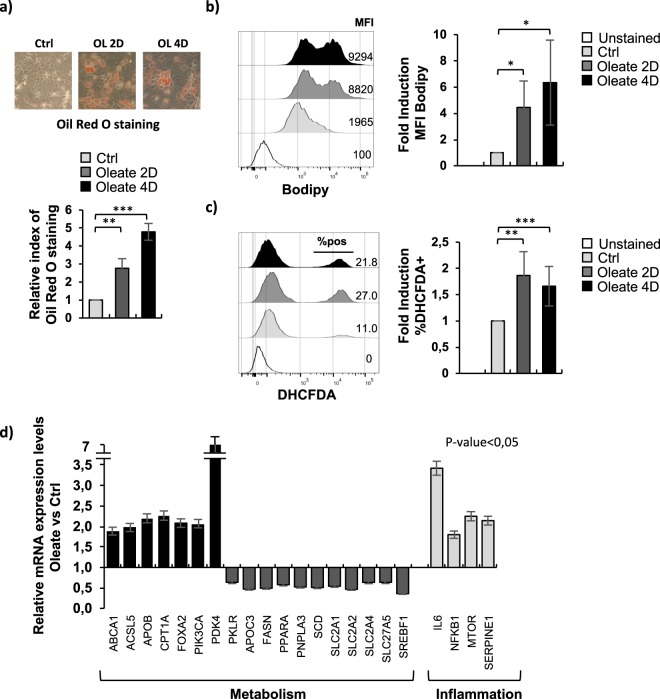
Sodium oleate treatment of dHepaRG cells induced lipid droplets accumulation, ROS formation and deregulated lipid metabolism and liver-specific genes expression. (**a**) Differentiated HepaRG (dHepaRG) cells were treated with vehicle (control) or with sodium oleate 250 μM for 2 or 4 days. After treatments, cells were stained with Oil Red O, lipid droplets are visible in red (Upper panels). Oil Red O dye was eluted and OD measured at 500 nm. Results are expressed as fold change of treated cells over control (Lower panel). (**b**) dHepaRG cells treated as in (**a**) were analyzed by citofluorimetry after Bodipy staining. Left panel: representative overlay of profile. Right panel: Histograms show MFI (Mean Fluorescence Intensity) as fold induction of treated cells over control from 3 independent experiments. (**c**) dHepaRG cells treated as in (**a**) were analyzed by citofluorimetry after DHCFDA (2′,7′-dichlorodihydrofluorescein diacetate) dye staining. Left panel: representative overlay of profile. Right panel: Histograms show % of DHCFDA positive cells as fold induction of treated cells over control from 3 independent experiments. (**d**) dHepaRG cells were treated as in (**a**) for 4 days. cDNAs were analyzed with a Human Fatty Liver RT² Profiler PCR Array, histograms show expression levels of a selected panel of genes as fold induction of treated cells over control. (Bars indicate S.D.; asterisks indicate p-value).

Human HepaRG cells exposed to 2% DMSO are able to differentiate into hepatocyte-like and biliary-like phenotypes and possess the ability to stably express liver-specific genes such as Albumin, AldolaseB, CYP2E1 and CYP3A4 (Supplementary 1a/b). Treatment of differentiated HepaRG cells (dHepaRG) with the fatty acid sodium oleate 250 μM for 2 or 4 days (Supplementary 1c) led to generation of cytoplasmic lipid droplets detected by Oil Red O staining (Fig. 1a), strongly increasing the cellular lipid content, as demonstrated by FACS analysis with Bodipy, a neutral lipid marker (Fig. 1b), and inducing cellular reactive oxygen species (ROS) generation, quantified by FACS analysis after 2′,7′-dichlorodihydrofluorescein diacetate (DHCFDA) staining (Fig. 1c). Since accumulation of triglycerides and ROS plays an important role in inducing hepatocyte injury associated with NAFLD^13^, we decided to investigate whether the effect of lipid droplets and ROS generation were able to trigger gene expression modulation in dHepaRG cells. We performed a qPCR analysis of sodium oleate 250 μM treated dHepaRG transcripts using a Human Fatty Liver Array (Sabiosciences), that profiles the expression of 84 key genes involved in the mechanisms of NAFLD and hepatic insulin resistance (Supplementary Table 1). We observed that several genes were significantly deregulated after treatment (Supplementary Fig. 2a/c). We showed in Fig. 1d (dark columns) that genes involved in carbohydrate (PDK4), cholesterol (ABCA1, APOB) or other lipid metabolism (ACSL5) were upregulated together with beta-oxidation genes (CPT1A) and insulin signaling pathway genes (FOXA2, PIK3CA). Interestingly, the glucose transporters (SLC2A1, SLC2A2, SLC2A4) and the fatty acid transport protein SLC27A5 were strongly reduced by sodium oleate treatment, together with the fatty acid biosynthesis gene SCD (Fig. 1d, dark grey columns). Moreover, key genes involved in the inflammatory response (IL6, NFKB1) and in the adipokine signaling pathway (SERPINE1, MTOR) were upregulated in response to lipid overload (Fig. 1d, light grey columns). These results demonstrated that sodium oleate treated human dHepaRG cells showed typical features of vesicular steatosis associated with a deregulation of gene expression.

### Fatty dHepaRG activate a cell autonomous IL6 inflammatory response

**Figure 2:**
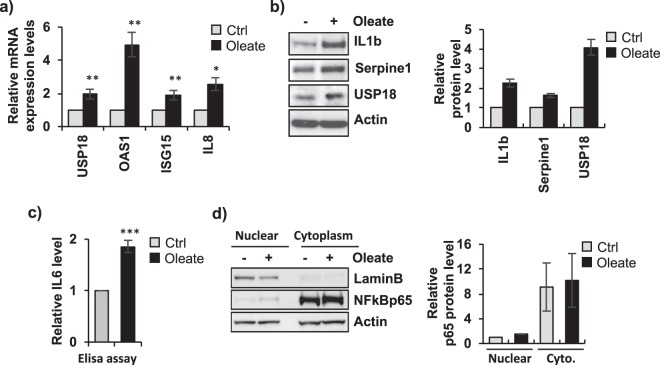
Fatty dHepaRG cells activate a cell autonomous IL6 inflammatory response. (**a**) dHepaRG cells were treated with vehicle (Ctrl) or with sodium oleate 250 μM for 4 days. cDNAs were analyzed by qPCR with primers specific for the indicated genes and normalized to Actin. (**b**) Left panel: total protein extracts were analyzed by Immunoblotting with the indicated antibodies. Right panel: densitometric analysis (ImageJ software). (**c**) Cells were treated as in a) and supernatants were colllected to quantify secreted IL6 levels by ELISA assay. (**d**) Left panel: nuclear and cytoplasmic protein extracts from cells treated as in a) were analysed by Immunoblotting with the indicated antibodies. Right panel: densitometric analysis (ImageJ software). Histograms show fold induction of treated cells versus control; bars indicate S.D.; asterisks indicate p-value. Full-length blots are included in Supplementary Fig. 9.

To investigate the role of the inflammatory response in the pathogenesis of vesicular steatosis, we analyzed a TaqMan Low Density Array (TLDA Cards) which profiles the expression of 96 key genes involved in the Interferon signaling pathway (Supplementary Table 1). We found deregulation of many genes after sodium oleate 250 μM treatment (Supplementary Fig. 2b/c), including upregulation of USP18, OAS1, ISG15 and IL8 transcripts (Fig. 2a). We could also observe an increase at the protein level of the inflammatory genes Serpine1, USP18 and IL1-b cytokine, activated by the inflammasome response (Fig. 2b), and an accumulation of the secreted cytokine IL6 (Fig. 2c). Interestingly, we observed that fatty dHepaRG cells did not activate a canonical NF-kB inflammatory pathway. Indeed, as demonstrated by Immunoblot analysis of nuclear and cytoplasmic protein extracts, NF-kB/p65 did not translocate into nuclei of sodium oleate treated cells (Fig. 2d).

**Figure 3:**
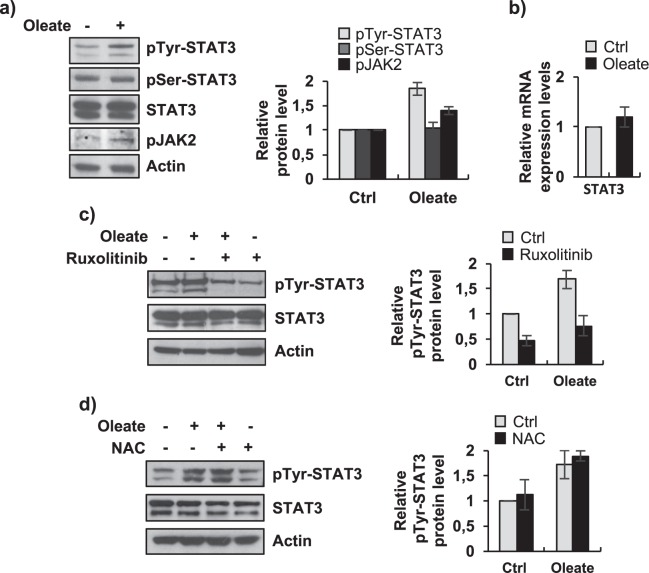
Activation of STAT3 in fatty dHepaRG. (**a**) Left panel: dHepaRG cells were treated with vehicle (Ctrl) or with sodium oleate 250 μM for 4 days, protein extracts were analyzed by immunoblotting with the indicated antibodies. Right panel: densitometric analysis (ImageJ software). (**b**) RNA transcripts were extracted from cells treated as in a) and cDNAs were analyzed with STAT3 specific primers and normalized to Actin. (**c/d**) dHepaRG cells were treated with vehicle (Ctrl) or with sodium oleate 250 μM for 4 days, and co-treated for the subsequent 18 hours with sodium oleate 250 μM and with Ruxolitinib 1 μM (**c**) or with sodium oleate 250 μM and NAC 10 mM (**d**). Left panels: protein extracts were analyzed by Immunoblot with the indicated antibodies. Right panels: densitometric analysis (ImageJ software). Histograms show relative protein level expressed as fold induction of treated cells versus control; bars indicate S.D.; asterisks indicate p-value. Full-length blots are included in Supplementary Fig. 10.

In order to assess if the observed IL6 accumulation lead to the activation of the JAK/STAT signaling, we evaluated phosphorylation levels of the down stream mediators of the pathway. After sodium oleate 250 μM treatment, STAT3-Tyr705 and JAK2 phosphorylation levels were significantly higher as compared to control cells, (Fig. 3a), whereas Ser727 phosphorylation of STAT3 and total STAT3 protein levels remained unchanged (Fig. 3a); STAT3 transcripts levels were also not affected (Fig. 3b). IL6/JAK2 pathway and ROS signaling are both described as STAT3 activators via Tyr705 phosphorylation^6,13^. To study whether these pathways are involved in STAT3 sodium oleate dependent Tyr705 phosphorylation, we treated cells with Ruxolitinib 1 μM, an inhibitor of JAK1/2 tyrosine kinases, and with the ROS inhibitor NAC 10 mM (N-acetylcysteine). We tested Ruxolitinib 1μM and NAC 10 mM activity by IL6 ELISA assay and DHCFDA FACS quantification, respectively (Supplementary Fig. 3a/b). We showed that Ruxolitinib was able to strongly reduce phospho-Tyr-STAT3 levels in both dHepaRG cells treated with sodium oleate 250 μM and control cells (Fig. 3c). Conversely, treatment with NAC 10 mM did not show any effect on both sodium oleate 250 μM and control phospho-Tyr-STAT3 levels (Fig. 3d). Taken together, these results indicated that fatty dHepaRG activate a cell-autonomous inflammatory response via STAT3-Tyr705 phosphorylation regulated by the IL6/JAK cascade, but not by the oxidative stress signaling.

### Identification of phospho-STAT3 target genes in fatty dHepaRG by a genome wide approach

**Figure 4:**
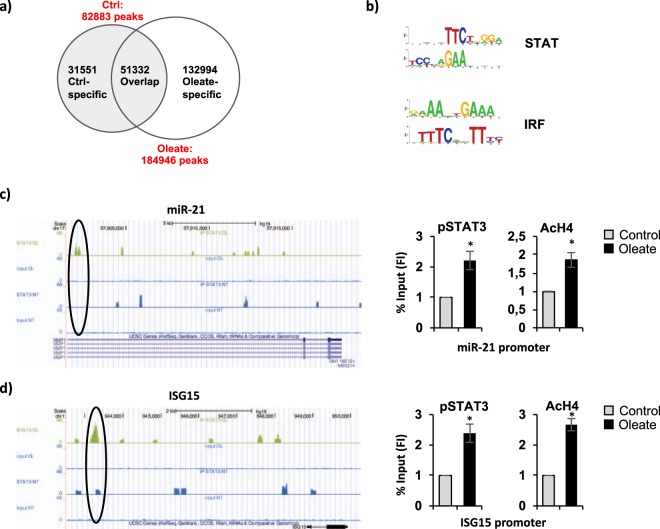
Identification of phospho-STAT3 binding sites in fatty dHepaRG by ChIP-seq analysis. dHepaRG cells were treated with sodium oleate 250 μM for 4 days (Oleate) or vehicle treated (Ctrl) and immunoprecipitated with a phospho-Tyr705-STAT3 specific antibody. (**a**) Candidate phospho-STAT3 binding sites from MACS2 peak calling (default MACS2 parameters, qvalue < 0.01). (**b**) Motif enrichment analysis: STAT (signal transducer and activator of transcription), IRF (interferon-regulatory factor). (**c/d**) Chip-seq profile (left panels) showing phospho-STAT3 enrichment (black circles) on miR-21 (**c**) and ISG15 (**d**) promoters after sodium oleate 250 μM treatment. Right panels: cross-linked chromatin from dHepaRG cells treated as in (**a**) was immunoprecipitated with a phosphoTyr705-STAT3 (pSTAT3) and an acetylated-Histone4 (AcH4) antibody and analyzed by qPCR with primers specific for the observated phospho-STAT3 peaks on miR-21 (**c**) and ISG15 (**d**) promoters, identified by chipseq analysis as shown in left panels. Histograms show Fold Induction (FI) of the % of Input (mean from 3 independent experiments; bars indicate S.D.; asterisks indicate p-value).

In order to identify, at a genome-wide level, genes potentially regulated by phospho-STAT3 during the pathogenesis of steatosis, we performed a Chromatin Immunoprecipitation Sequencing (ChIP-seq) analysis on fatty dHepaRG cells using a phosphoTyr705-STAT3-specific antibody. Raw data for ChIP-seq are available at GEO (GSE89157). Results indicated 82883 total peaks, corresponding to phospho-STAT3 candidate binding sites, in control dHepaRG cells (Ctrl) and 184946 in sodium oleate 250 μM treated dHepaRG cells, which partially overlapped with the control peaks (51332 peaks); among the 184946 peaks in sodium oleate treated dHepaRG cells we found a significant amount of phospho-STAT3 bindings specifically induced by sodium oleate (132994 Oleate-specific) (Fig. 4a). Motif enrichment analysis associated to ChIP-Seq peaks identified STAT and IRF (interferon-regulatory factor) transcription factors putative binding sites (Fig. 4b). Automatically scanning for phospho-STAT3 binding sites revealed that several miRNAs promoters are bound in sodium oleate-treated cells, including miR-21 (Fig. 4c left panel) and miR-122 (Supplementary Fig. 4). Moreover, phospho-STAT3 has been found also on promoters of several metabolic and inflammatory genes, including ISG15 (Fig. 4d left panel), PLIN4 and USP18 (Supplementary Fig. 4). To validate these results, we performed an independent ChIp assay against acetylated-Histone4 and phosphoTyr705-STAT3 in dHepaRG cells treated with sodium oleate. As shown in Fig. 4c,d (right panels), we confirmed phospho-STAT3 enrichment on miR-21 and ISG15 promoters in response to lipid accumulation, which paralleled with increased acetylation levels of bound Histone-4, a marker of transcriptional active chromatin. Overall, these results showed that phoshpo-STAT3 is induced to bind a panel of genes and miRNAs in fatty dHepaRG cells, suggesting a role in the pathogenesis of liver steatosis.

### A STAT3-microRNA signaling is activated during lipid accumulation

**Figure 5:**
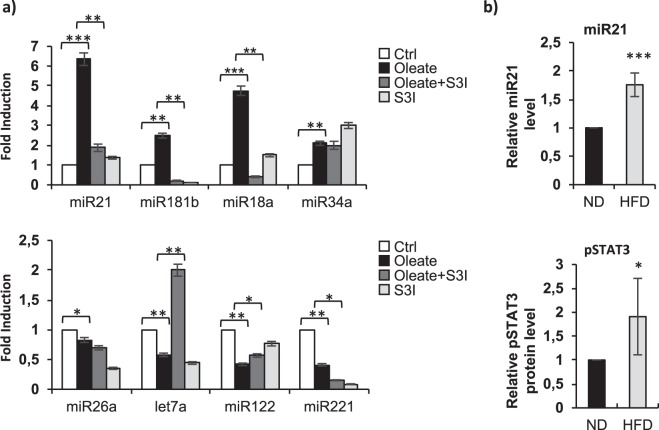
A STAT3-microRNA signaling is involved in lipid accumulation. (**a**) dHepaRG cells were treated with sodium oleate 250 μM for 4 days or vehicle treated (Ctrl) and co-treated for the subsequent 48 hours with sodium oleate 250 μM plus S3I-201 100 μM or S3I-201 alone. Total RNA were extracted and miRNAs levels were analyzed by qPCR (TaqMan MicroRNA Assay, Applied Biosystems), samples were normalized to the RNU38B endogenous control. Histograms show mean value expressed as fold induction of treated versus control cells. (**b**) C5BL6 mice were fed a high-fat diet (HFD) or normal diet (ND) for 16 weeks. Upper panel: total RNA were extracted from mice liver and q-PCR quantification of miR-21 expression was normalized to the snoRNA234 endogenous control. Lower panel: densitometric analysis (ImageJ software) of pSTAT3 protein levels from liver mice total protein extracts analyzed by immunoblotting. Histograms show mean value expressed as fold induction of HFD samples versus ND samples. (Bars indicate S.D.; asterisks indicate p-value). Immunoblot is available in Supplementary Fig. 6e.

We selected a panel of known STAT3-related miRNAs and miRNAs deregulated in NAFLD/NASH, as shown in Table 1. We observed that dHepaRG sodium oleate (250 μM) dependent lipid accumulation led to a differential expression of the selected hepatic/STAT3-responsive miRNAs (Fig. 4a): in particular, a significant increase of miR-21, miR-181-b, miR-18a and miR-34a levels (Fig. 5a upper panel, black columns) and a significant downregulation of miR-26a, let7a, miR-122, and miR-221 (Fig. 5a lower panel, black columns). To study the role of STAT3 in the regulation of these miRNAs we treated dHepaRG cells with a STAT3 inhibitor, S3I-201, which targets STAT3 DNA-binding and transcriptional activities^14^. We tested S3I-201 inhibitory activity by IL6 ELISA assay and qPCR mRNA levels quantification of a known STAT3-transcritpional target HP (Haptoglobin) (Supplementary Fig. 5a/b). Interestingly, co-treatment with sodium oleate 250 μM and with S3I-201 100 μM, was able to significantly reduce sodium oleate dependent upregulation of miR-21, miR-181-b, miR-18a expression (Fig. 5a upper panel, dark grey columns), partially restored miR-122 expression and strongly increased let7a levels (Fig. 5a lower panel, dark grey columns). These results demonstrated that phospho-STAT3 regulates the expression levels of a panel of miRNAs, including miR-21, in response to lipid accumulation. We focused on miR-21 for its emerging role in lipid metabolism and recent observations of increased expression in hepatocellular carcinoma and in NAFLD patients^15–17^. To confirm a direct role of STAT3 on miR-21 transcription we demonstrated that pri-miR-21 expression level correlates with mature-miR-21 regulation (Supplementary Fig. 5c).

**Table 1:** NAFLD/NASH and/or STAT3 regulated miRNA.

	Upregulated miRNAs	Downregulated miRNAs	References
NAFLD/NASH	miR-18a, miR-21, miR-34a, miR-122	miR-26a, miR-122, miR-221	Pirola C. J., *et al*.^6^; Gerhard G. S.^8^
STAT3	miR-21, miR-18a		Cao Q.^10^; Jin K.^11^; Iliopoulos D.^31^

To investigate *in vivo* miR-21 expression levels in a mouse model of liver steatosis, we took advantage of C57/BL6 mice fed a High-Fat diet (HFD). HFD mice showed typical features of NAFLD/NASH, with increased ALT (alanine aminotransferase) liver enzyme activity levels, body weight and glucose levels as compared to mice fed normal diet (ND) (Supplementary Fig. 6a–d). We observed that miR-21 was overexpressed also in HFD mice liver as compared to ND mice (Fig. 5b, upper panel) and phospho-STAT3 protein levels are upregulated (Fig. 5b, lower panel and Supplementary Fig. 6e).

### STAT3 inhibition reduced dHepaRG sodium oleate-dependent lipid accumulation

**Figure 6:**
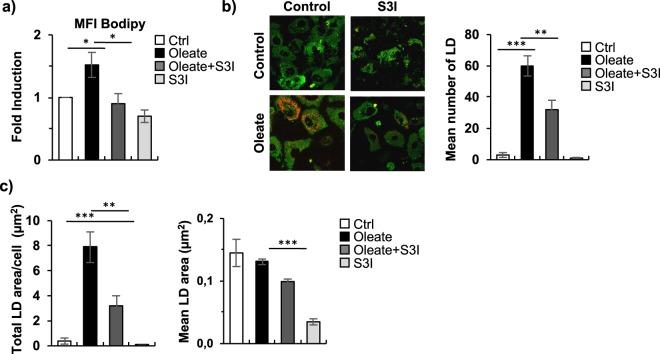
STAT3 inhibition reduced dHepaRG sodium oleate-dependent lipid accumulation. (**a**) FACS analysis of dHepaRG cells treated as in Fig. 5(a) and stained with the Bodipy lipid probe. Histograms show fold change of treated samples over controls of Mean Bodipy Fluorescent Intensity (MFI). (**b**) CARS analysis of dHepaRG cells treated as in Fig. 5(a). Left panels: representative images showing lipid droplets fluorescence in red. Right panel: histogram shows mean number of LD, bars show S.E. (**c**) CARS analysis of dHepaRG cells treated as in Fig. 5(a), histograms show total droplet area/cell (left) and mean droplet area (right). (Bars indicate S.E.; asterisks indicate p-value).

In addition to transcriptionally regulating a panel of miRNAs, we investigated whether STAT3 activation could be directly involved in the induction of vesicular steatosis. By FACS analysis after Bodipy staining, we showed that inhibition of STAT3 activity with S3I-201 100 μM strongly reduced lipid accumulation in sodium oleate 250 μM co-treated dHepaRG cells as compared to the sodium oleate treatment alone (Fig. 6a). Similar results were observed after co-treatment with Ruxolitinib 1 μM, which inhibits STAT3 phosphorylation and slightly but significantly reduced Bodipy MFI (Supplementary Fig. 7a). Conversely, inhibition of ROS generation by co-treatment with NAC 10 mM did not affect lipid accumulation induced by sodium oleate 250 μM (Supplementary Fig. 7b). Moreover, we showed that STAT3 inhibition by S3I-201 reverted oleate dependent up-regulation of lipid metabolism genes, analysed by qPCR (Supplementary Fig. 7c). To further characterize the role of STAT3 in lipid droplets generation we made use of the nonlinear Coherent Anti-Stokes Raman Scattering (CARS) microscopy^18,19^. Treatment with sodium oleate 250 μM induced a significant increase in the number of LD (Fig. 6b), which led to a higher total droplets area per cell (Fig. 6c, left panel), but an unchanged mean LD area (Fig. 6c, right panel), indicating that after oleate treatment LD increase in number but not in dimension. Interestingly, co-treatment with STAT3 inhibitor S3I-201, dramatically decreased sodium oleate induced LD number (Fig. 6b) and total LD area per cell (Fig. 6c, left panel), whereas it slightly decreased mean LD area (Fig. 6c, right panel).

Therefore, these results strongly demonstrate that STAT3 activation directly contributes to lipid accumulation and LD generation and is able to drive transcription of several metabolism genes.

### Inhibition of phospho-STAT3 and miR-21 expression by metformin long-term therapy protected mice against age dependent liver steatosis

**Figure 7:**
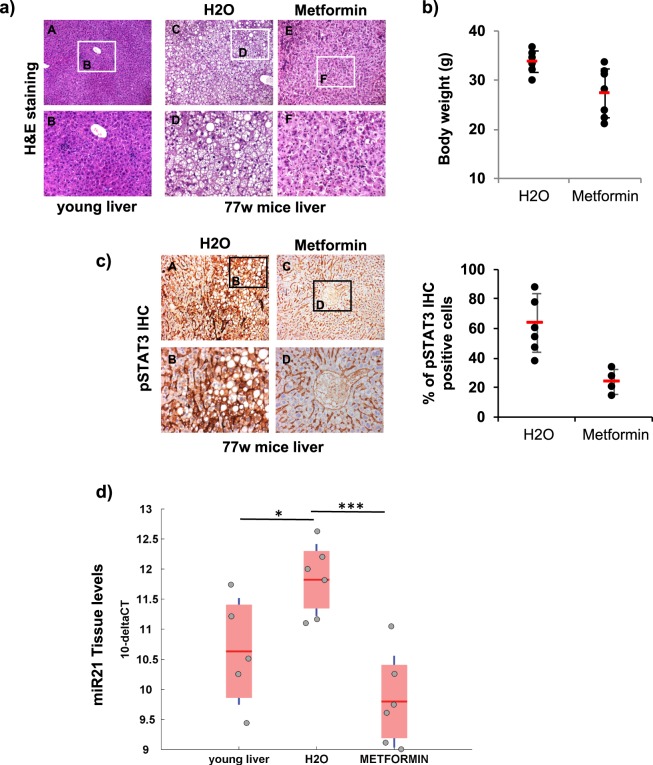
Inhibition of phospho-STAT3 and miR-21 expression by metformin long-term therapy protected mice against age dependent liver steatosis. (**a**) 5 weeks aged C57/BL6 mice were daily treated with either water or metformin (50 mpk) for 72 weeks. At the end of treatment (77 weeks) FFPE livers were stained with Hematoxylin/Eosin. Images A-B: 5 weeks aged mice before treatment; images C-D: 77 weeks old control mice (H20); images E-F: 77 weeks old mice metformin treated. (**b**) Dot plot showing body weight of mice treated as in (**a**). (**c**) Phospho-STAT3 immunohistochemistry (IHC) on liver sections of mice treated as in (**a**). Left panels: positive IHC cells are stained brown color, images A-B control mice (H20); images C-D metformin treated mice. Right panel: % number of phospho-STAT3 positive cells (6 fields for 4 non-consecutive stained sections per sample were scored). **d**) Boxplot representing q-PCR quantification of miR-21 expression in liver tissue from 77 weeks mice treated as in (**a**) (H_2_0 and Metformin) and 5 weeks mice (young) at the beginning of treatment. SnoRNA202 was used as endogenous control to standardize miRNAs expression. Results are expressed as 10^−DCt^ (Asterisks indicate p-value).

To evaluate STAT3 and miR21 implication in liver steatosis also *in vivo*, we treated 5 weeks aged C57/BL6 mice for 72 weeks with metformin, an established anti-diabetic drug, that is able to modulate STAT3 signaling by repressing IL-6-dependent phosphorylation of STAT3^20,21^. At sacrifice, 77 weeks old control mice (H_2_O treated) showed an evident macro-vesicular liver steatosis due to aging, with well-defined fat droplets occupying the cytoplasm of hepatocytes (Fig. 7a, panels C,D) as compared to samples collected from young 5 weeks old mice (Fig. 7a panels A,B). Interestingly, metformin-treated mice showed a significantly attenuated liver steatosis (Fig. 7a panels E,F) as compared to control mice (H_2_O) (Fig. 7a, panels C,D). Moreover, metformin treatment reduced mice body weight (Fig. 7b) and decreased number of phospho-STAT3 positive hepatocyte cells in mice liver (Fig. 7c, left panel), as shown by Immunohistochemistry, with an average of 63.8% in control mice (H_2_O) versus only 23.8% in metformin treated mice (Fig. 7c, right panel). In addition, miR-21 levels were significantly downregulated in liver tissue from mice treated with metformin as compared to control and young 5weeks mice (Fig. 7d) and also in mice serum (Supplementary Fig. 8), paralleling phospho-STAT3 expression levels.

These results demonstrate that downregulation of both phospho-STAT3 and miR-21 levels in the liver after long-term treatment with metformin improves age-dependent macro-vesicular liver steatosis in mice.

## Discussion

NAFLD is a complex disease trait whose development and progression are determined by the combination of genetic and epigenetic changes^1,4,8,9^. NAFLD and NASH are associated with substantial metabolic stress and inflammation, mediated by IL6/STAT3 signaling, that affects both cell survival and determine an increased risk of developing hepatocellular carcinoma^3,6,22^.

The present study demonstrates the activation of the IL6-STAT3 axis in an *in vitro* model of vesicular steatosis, with consequent de-regulation of several STAT3-related miRNAs. Human hepatic HepaRG cell line, a valuable alternative to primary human hepatocytes, has been used as a model of vesicular steatosis^23^. Indeed, we already showed that addition of sodium oleate to differentiated HepaRG cells induces lipid storage in droplets, that were statistically quantified in terms of numbers, distribution and morphology at single cell level, utilizing a new label-free microscopy technique, coherent anti-Stokes Raman scattering (CARS)^12^. CARS microscopy it is now recognized as a powerful non-invasiveness tool for the investigation of biological specimens, as it is capable of imaging unstained living cells with a high vibrational sensitivity and chemical selectivity^18,19^.

Here, we demonstrated that lipid overloaded dHepaRG cells induced the activation of an intracellular autonomous inflammatory response, with increased STAT3-Tyr705 phosphorylation levels. Contrarily, STAT3-Ser727 phosphorylation, which also enhances STAT3 transcriptional efficacy, but it is not mandatory for STAT3 activation^24^, was not modulated. We found by Chip-seq using an antibody that specifically recognizes phosphorylation at specific Tyrosine 705 residue, that phospho-STAT3-Tyr705 is able to differentially bind many promoters, including miR-21 regulatory region, after the induction of vesicular steatosis in dHepaRG cells (raw data for ChIP-seq are available at GEO GSE89157). Lipid overloading-dependent activation of STAT3 is directly responsible for the transcriptional modulation of miR-21 and several other miRNAs. Indeed, we were able to show for the first time that inhibition of STAT3 activity deregulates miRNA expression and in parallel improved steatohepatitis *in vitro* and *in vivo*, reducing neutral lipid accumulation and droplets generation, suggesting STAT3 as a potential therapeutic target for NAFLD.

No drug is currently available as a specific treatment for NAFLD and the combination of diet and lifestyle modifications remain the pillars of NAFLD and NASH management^25,26^. Nevertheless, it has been shown that metformin has anti-oxidant, anti-inflammatory and anti-fibrogenic properties in liver diseases and induces reduction in tissue lipid storage with an increase in both fatty acid oxidation and inhibition of lipogenesis, presumably mediated by AMPK activation^27–29^. Moreover, metformin is able to induce alterations in STAT3 signaling in many cell types^20,21^. However, metformin-induced activation of energy metabolism, via the energy-sensor AMPK, may not account for all benefit effects of the drug on NAFLD and the exact molecular mechanisms of its therapeutic action remain obscure^27,29^. Here, we have been able to show that reduction of STAT3 activation and miR-21 levels in C57/BL6 mice liver, by metformin long-term treatment (72 weeks), strongly ameliorated steatohepatitis that spontaneously occurred in control mice due to aging.

Altogether, the results we obtained *in vitro* and *in vivo* converge to indicate that miR-21 is involved in NAFLD pathogenesis. Indeed, it has been recently shown, in mice fed a high-fat diet, that liver-specific miR-21 knockout prevents steatosis by altering the expression of several master metabolic regulators^30^, and in a model of NASH-associated liver damage, miR-21 knockout mice display reduced steatosis, inflammation and lipoapoptosis, with impairment of fibrosis^15^. MiR-21 is overexpressed in many human cancers and is thought to play a significant role in carcinogenesis^31,32^. Circulating and liver tissue miR-21 level are also significantly increased in patients suffering from NASH and in inflammatory states of fatty liver disease^15–17^. However, the mechanism by which miR-21 might contribute to NASH/NAFLD development is still unknown. Bio-informatic prediction of miR-21 potential mRNAs target revealed putative genes involved in both HCC and in Fatty Liver disease (Table 2), including PTEN^33^, and SLC2A1 and PPARA that also showed reduced expression after sodium oleate treatment in dHepaRG cells (Fig. 1d). This analysis suggests a role for miR21 in fine tuning metabolic processes in hepatocytes by regulating gene expression at the post-transcriptional level.

**Table 2:** *In silico* analisys of miR-21 predicted target genes in HCC and Fatty Liver disease (mirwalk2.0).

HCC	HCC and FATTY LIVER	FATTY LIVER
AKT2, BASP1, BCL2, CCL20, CCNG1, CCR1, CCR7, CD47, CDC25A, CDK6, CLOCK, EDIL3, EGFR, ERBB2, FANCC, FAS, FASLG, FGF18, FUBP1, IL12A, IL1B, JAG1, KLK10, LIFR, MATN2, MEF2A, MEF2C, MMP2, MMP9, MSH2, MTAP, MUC1, MYC, NCOA3, NFKB1, PARP1, PDCD4, PER3, PIK3C2A, PIK3R1, PTEN, PTK2, RB1, RDH11, RPL36A, RTN4, SKP2, SLC2A1, SP1, SPDYA, SPRY2, STAT3, TGFBI, TGFBR3, TIAM1, TP53BP2, TP63, WNT5A	E2F1, ICAM1, PPARA, TGFB1, VEGFA	CLOCK, EIF2S1, LUM, TNFRSF11B

In conclusion, here we described for the first time an integrated signaling module that links liver steatosis and the activation of IL6/STAT3 signaling with downstream STAT3-dependent activation of a number of miRNAs, including the onco-mir miR-21. STAT3 activation is responsible for the inflammatory micro-environment that facilitates lipid accumulation leading to the dysregulation of several miRNAs. The observation that down-regulating by metformin the activation of STAT3 and the overexpression of miR-21 *in vivo* limited lipid accumulation and liver damage identifies miR-21 and STAT3 inhibition as new promising pharmacological targets for treating NAFLD. Indeed, even though in pilot studies metformin was shown to improve fatty liver disease in mouse models of NAFLD^34^, as well as in NAFLD/NASH patients^28,29^, American guidelines do not recommend metformin for the treatment of adult NAFLD^27^.

On the other hand, several small molecules that *indirectly* inhibit STAT3 activation, by targeting the binding of cytokines and growth factors to their receptors, have been developed and some of them are currently undergoing clinical application to treat human diseases, including cancer^35^. Nevertheless, these agents have suboptimal potency and pharmacokinetic parameters, and/or poorly defined mechanisms, including S3I-201^36^. A number of *direct* STAT3 inhibitors molecules have been developed to treat cancer and other human diseases, but are currently at preclinical stage^35,37^. Therefore, directly targeting STAT3 still remains an important aim to reduce off-target effects for clinical application, potentially including NAFLD management.

## Material and Methods

### Cell Culture and treatments

Human hepatic HepaRG cells were seeded at low density in William’s E medium with GlutaMAX (Gibco), supplemented with 10% FBS (Hyclone II GE), 1% penicillin/streptomycin (Sigma), 5 µg/mL insulin (Sigma), 0.5 µM hydrocortisone hemisuccinate (Sigma). After 1 week at confluence, cells were shifted into the same medium supplemented with 50 µM hydrocortisone hemisuccinate and 2% DMSO (Sigma) for 2 more weeks to obtain confluent differentiated cultures. Cells were then treated with different drugs (Supplementary Fig. 1c) for the indicated time at the following final concentrations: sodium oleate 250 μM (Sigma); S3I-201 100 μM alias NSC74859 (Selleckchem.); N-acetylcisteina 10 mM (Sigma); Ruxolitinib 1 μM (Selleckchem.). Cytoxicity of compounds was tested by MTT assay (Supplementary methods and Supplementary Fig. 1d).

### Animal model

C57/BL6 mice were chronically treated with either water or metformin (50 mpk) for 72 weeks (7 mice per group) (Fig. 7), or they were fed with either normal diet or with a High-Fat Diet (59% fat, 15% protein, 26% carbohydrate) for 16 weeks (7 mice per group) (Fig. 5b). At the end of the study, livers from metformin treated mice were fixed in buffered formalin and embedded in paraffin, while livers from HFD fed mice were nitrogen frozen. Information about animal conditions are described in Supplementary methods. All methods were carried out in accordance with relevant guidelines and regulations and all experimental protocols were approved by the Italian Ministry of Health.

### Immunohistochemistry

5 μm-thick sections were trimmed from each FFPE histological specimen, and stained with Haematoxylin/Eosin and with phospho-STAT3 (Tyr705) (XP Cell Signaling, #9145) antibody as described in Supplementary methods.

### FACS analysis

To quantify lipid accumulation Bodipy dye (Sigma) was used (100 nmol/L, 40′, 37 °C, excitation/emission wavelengths 505/515 nm). 2′,7′-Dichlorofluorescin diacetate (DHCFDA) dye (Sigma) was used to monitor intracellular ROS production (10 μmol/L, 20′, 37 °C, excitation/emission wavelengths 488/520 nm). Cytofluorimetric analysis was performed using a FACS-CANTO (BD).

### ELISA assay

The expression levels of interleukin 6 (IL6) secreted from sodium oleate treated dHepaRG cells were detected by enzyme-linked immunosorbent assay, IL6 ELISA, from Abcam (Ab 46042). Cell culture media was centrifuged at 1,000 g for 10 minutes to remove debris and supernatants were collected to perform standard ELISA as manufacturer’s protocol.

### RNA and miRNAs extraction and analysis

See Supplementary methods.

### Immunoblotting

Cells were lysed in NET buffer (50 mM Tris–HCl pH 7.5, 150 mM NaCl, 0.1% NP-40, 1 mM EDTA pH 8) and immunoblotted with the following antibodies: anti-STAT3 (#9139), anti-phospho-STAT3(Tyr705) (#9131), anti-phospho-STAT3(Ser727) (#9134), anti-IL1β (#12242), anti-phospho-JAK2(Tyr1007/1008) (#3771), anti-PAI-1 (D9C4) (#11907) from Cell Signaling; anti-NFκB p65 (sc-372), anti-USP18 (sc-98431) and anti-Actin (sc-1616) from Santa Cruz Biotechnology. Proteins of interest were detected with HRP-conjugated anti-mouse/rabbit/goat IgG antibodies from Santa Cruz Biotechnology and visualized with the Pierce ECL Western blotting substrate (ThermoScientific), according to the provided protocol. Autoradiography images were developed with a KODAK MIN-R processor. Full-length blots are included in Supplementary Figures 9/10.

### ChIP and ChIPseq

Chromatin from dHepaRG cells was immuno-precipitated with the following antibodies: anti-phospho-STAT3-Tyr705 (XP Cell Signaling, #9145), anti-tetra-acetylated-histone-H4-Lys-6-9-13-17 (06-866 Upstate) and non-specific IgG (Santa Cruz Biotechnology Inc.). Chromatine Immunoprecipitated was analyzed by qPCR using fluorescent dye SYBR Green in a Light Cycler 480 instrument (Roche Diagnostics). Deep sequencing analysis^38,39^ was performed as described in Supplementary methods.

### CARS microscopy

A multimodal nonlinear microscope was used to record images in formaldehyde fixed dHepaRG cells, using the strong methylene vibration at 2840 cm^−1^ as coherent Raman image contrast for lipids, as well as multiphoton auto-fluorescence contrast, as described in Supplementary methods. All data generated by CARS are included in Supplementary Table 2.

### Statistics

P-values were determined using the 2-tailed Student’s T-test: *0,01 ≤ P < 0,05; **0,001 ≤ P < 0,01; ***P < 0,001.

## Electronic supplementary material

Supplementary Information

## Data Availability

The datasets generated by Chipseq and analysed during the current study are available in the GEO repository data (GSE89157).
