# Supplementary material for: Targeting a phospho-STAT3-miRNAs pathway improves vesicular hepatic steatosis in an in vitro and in vivo model
Source: Sci Rep. 2018 Sep 11;8:13638. doi: 10.1038/s41598-018-31835-2 (PMC6134080; doi:10.1038/s41598-018-31835-2)
Supplement: Supplementary file 1 — Supplementary Information [file 41598_2018_31835_MOESM1_ESM.pdf]

SUPPLEMENTARY INFORMATION

Targeting a phosphoSTAT3-miRNAs pathway improves vesicular hepatic steatosis in an *in vitro* and *in vivo* model.

Laura Belloni[1,#], Silvia Di Cocco[2,3,#], Francesca Guerrieri[1], Abigail D. G. Nunn[1], Silvia Piconese[2,4], Debora Salerno[1], Barbara Testoni[5], Claudio Pulito[6], Federica Mori[6], Matteo Pallocca[7], Andrea Sacconi[8], Elisa Vivoli[9], Fabio Marra[9], Sabrina Strano[6,10], Giovanni Blandino[8], Massimo Levrero[1,2,5] and Natalia Pediconi[1,3*]

[1] Center for Life Nano Science@Sapienza, Istituto Italiano di Tecnologia, Rome, Italy
[2] Department of Internal Medicine - DMISM, Sapienza University, Rome, Italy
[3] Department of Molecular Medicine, Sapienza University, Rome, Italy
[4] Pasteur Institute Italy-Fondazione Cenci Bolognetti, Rome, Italy
[5] Centre de Recherche en Cancérologie de Lyon (CRCL), UMR 1052 - CNRS 5286
[6] Molecular Chemoprevention Group, National Cancer Institute Regina Elena, Rome, Italy
[7] UOSD SAFU, National Cancer Institute Regina Elena, Rome, Italy
[8] Oncogenomic and Epigenetic Unit, National Cancer Institute Regina Elena, Rome, Italy
[9] Dipartimento di Medicina Sperimentale e Clinica, Università di Firenze, Italy
[10] Department of Oncology, Juravinski Cancer Center-McMaster University Hamilton, ON, Canada

[#] These authors contributed equally to this work.
***Corresponding author**: Natalia Pediconi, viale Regina Elena 291, 00161 Rome, Italy, tel+39 0649255664, natalia.pediconi@iit.it

TABLE OF CONTENTS:

- Methods
- Figures
- Tables

SUPPLEMENTARY METHODS

MTT assay. Assessment of cytotoxicity was performed by adding a small amount of the CellTiter 96® AQueous One Solution Reagent (Promega #G3582) directly to culture, incubating for 1–4 hours and then recording absorbance at 490nm with a 96-well plate reader, according to manufacturer's instruction.

Oil red O Staining. Neutral lipid accumulation was determined by Oil Red O staining. A stock solution of Oil Red O (Sigma) was prepared in isopropanol (0.35gr/100ml). After each treatment, cells were fixed in 4% formaldehyde, washed with 60% isopropanol and incubated at RT for one hour with Oil Red O-saturated solution (stock solution:water 3:2). Oil Red O dye was eluted by adding 100% isopropanol and incubate for 10 min and measure OD at 500 nm using 100% isopropanol as blank.

Animal model.

HFD fed mice. Male C57/BL6 mice (20–25 g) were used. Ten mice were housed per cage (26 × 41 cm). The cages were placed in the experimental room 24 h before the test for acclimatization. The animals were fed a standard laboratory diet or High-Fat diet (59% fat, 15% protein, 26% carbohydrate) and tap water ad libitum and kept at 23 ± 1°C with a 12 h light/dark cycle, light on at 7a.m. Animals were naive and used only once.

Metformin treated mice. Sex Gender: male and female five week old. Metformin (16 mg /Kg) were administered in drinking water for 24 hours one day per week for 78 weeks. Mice were housed inside cages with paper filter covers; bedding are sterilized. Animals were housed under a light-dark cycle, keeping °C temperature 55±10% relative humidity, about 15-20 filtered air changes/hour and 12 hour circadian cycle of artificial light (7 a.m., 7 p.m.). Each mouse was offered daily a complete pellet diet (GLP4RF21, Mucedola) throughout the study. All animals were weighed during the whole treatment period in order to calculate the percent bodyweight loss during experimental period. A body weight loss (BWL) ≥ 10% has been considered as sign of suffering and involved the mice sacrifice. The authorization to use animals were obtained by the Italian Health Authority. The Care and Husbandry of animals are in accordance with European Directives no. 86/609 and with the Italian Regulatory system (D.L. vo no. 26/14). All methods were carried out in accordance with relevant guidelines and regulations and all experimental protocols were approved by the Italian Ministry of Health. All efforts were made to minimize animal suffering and to reduce the number of animals used.

Immuno-histochemistry. 5µm-thick sections were trimmed from each FFPE histological specimen and stained with Haematoxylin/Eosin and with phospho-STAT3. Briefly, tissue slides were dewaxed and endogenous peroxidase was blocked by 3% hydrogen peroxide. After blocking with normal goat serum, primary antibody against phospho-STAT3 (Tyr705) (XP Cell Signaling, #9145) was applied to the tissue slides, followed by a specific secondary antibody. The detection system defines positive IHC as brown color stains. 6 fields for 4 non-consecutive stained sections per sample were scored and the number of positive cells assigned (double-blind control procedure).

RNA extraction and analysis. Total RNAs from HepaRG cells were isolated using TRIzol reagent (Invitrogen). cDNA was synthesized using a Maxima-H-minus-First-Strand-cDNA Synthesis Kit (Thermoscientific) and analysed with gene specific primers by qPCR using the fluorescent dye SYBR Green. cDNA were also analysed with a RT² Profiler™ PCR Array Human Fatty Liver (cat.PAHS-157Z, Sabiosciences,) in a Light Cycler 480 instrument (Roche Diagnostics). To perform Taqman® Low Density Array (TLDA; Applied Biosystems) analysis, Superscript® VILO™ cDNA synthesis kit (11754, Life Technologies) was used for retro-transcription and each TLDA plate was loaded with a cDNA amount corresponding to 100 ng of initial RNA per lane and run in a 7900HT Fast Real-Time (Applied Biosystems) according to manufacturer's instructions. Data were collected using SDS 2.3 software and analyzed with RQ Manager 1.2 and StatMiner programs (Applied Biosystems). 18s was used as internal control for normalizing equal loading of the samples. Complete list of genes included in the TLDA and Fatty Liver card are listed in Supplementary Table 1.

miRNA extraction and analysis. Mature miRNAs were extracted from cells with TRIzol reagent (Invitrogen) or from mice FFPE samples using miRNeasy FFPE (Qiagen). q-PCR quantification of miRNA and pri-miRNA expression was performed using TaqMan MicroRNA Assays (Applied Biosystems). Relative expression was calculated using the comparative Ct method (2-ΔCt, ΔCt=Ct(Target gene)-Ct(housekeeping gene)). 200 µl of blood was collected from anesthetized mice by retro-orbital puncture using citrate 3.8% as anti-coagulant (1/10 Volume) and cell-free total RNA from serum was extracted using the miRNeasy Serum/Plasma Kit (Qiagen). The first-strand cDNA was synthesized with miScript Reverse Transcription Kit (Qiagen). qPCR assays were performed by STEPOne PCR (Agilent) using the miScript SYBR Green PCR Kit (Qiagen). hsa-miR-21-5p miScript primer Assay was used. The C. Elegans miR-39 (miRNeasy Serum/Plasma Spike-In Control, Qiagen) was used as control to standardize miRNA expression.

ChIPseq. Immunoprecipitated chromatin from dHepaRG cells was purified by

phenol/chloroform (1:1) extraction associated to Phase Lock Gel (5 Prime) and ethanol precipitation. ChIPed chromatin is processed according to the Illumina ChIp-Seq libraries generation protocol (IP-102-1001) and sequenced on Illumina Hiseq 1500 platform. The eluate was quantified by using a Qubit (Invitrogen) fluorometer. DNA fragments recovered from reverse cross-linked chromatin are repaired, ligated to adapters, size selected and PCR-amplified to generate NGS libraries following the Illumina DNA Library Construction Kit (IP-102-1001). Raw reads were aligned to HG19 reference genome with Bowtie2 (38). MACS2 (39) was used to call peaks with default calling parameters (qvalue<0.01) and --bdg flag to generate bigWig files (bdgcmp command). Custom python scripting enabled intersection of pSTAT3 peaks with miRs promoters coordinates. Coordinates for miRs promoters were obtained by downloading miRs transcription starting sites from UCSC hgTable website and considering a 20kb upstream window. Raw data for ChIP-seq are available at GEO (GSE89157).

CARS microscopy. Coherent Anti-Stokes Raman Scattering (CARS) microscopy allows chemically specific label-free image contrast with up to pico(10^{-12})-gram sensitivity and sub-micron spatial resolution, avoiding problems that can be associated with the use of perturbatively large fluorescent labels or stains which is particularly pertinent for relatively smaller lipid molecules (16,17). CARS microscopy offers significant speed advantages over spontaneous Raman microscopy in that it generates a much-enhanced signal (5-10 orders of magnitude) due to the coherent driving of the molecular vibration by a pair of intense, short laser pulses. A multimodal nonlinear microscope was used to record images in fixed cells, using the strong methylene vibration at 2840cm^{-1} as coherent Raman image contrast for lipids, as well as multiphoton fluorescence. The experiment consists of a picosecond laser system (Levante Emerald OPO, by APE Angewandte Physik & Elektronik GmbH, Germany, pumped by Coherent Plecter laser at 532 nm) coupled to a commercial inverted laser scanning microscope (Olympus IX83 with FV1200MPE multiphoton scan unit). In brief, the laser system generates two 6 ps duration pulses at 80MHz repetition rate, the pump (817nm) and Stokes (1064nm) beams, that are spatially and temporally overlapped and then coupled into the scan unit of the microscope. Combined power of the two beams was fixed throughout the experiment, and estimated to be 50mW at the sample. A 100×/NA=1.3 oil immersion objective was used to focus the light for imaging over a square field of 127μm with lateral pixel size 124nm (1024×1024 pixels). The pixel dwell time was 20μs. The CARS signal in the epi (back-reflected) direction was collected using one PMT channel of the microscope's 2-channel fluorescence detection unit, with the entrance to the PMT modified

by the substitution of a broad bandwidth red fluorescence filter for a specific filter for the 2840 cm^{-1} band, detecting at 663 nm (Semrock FF01-661/20). Complementary multiphoton fluorescence images, emitted in the green part of the visible spectrum, and detected also in epi, from excitation with two-photons at 817 nm, were recorded simultaneously with the CARS images, using the other PMT channel. All images processing and analysis was carried out using the Fiji implementation of ImageJ. CARS and Multiphoton fluorescence images were treated for noise removal using the “Despeckle” function before combining in composite images.

SUPPLEMENTARY FIGURES

Supplementary Fig. 1

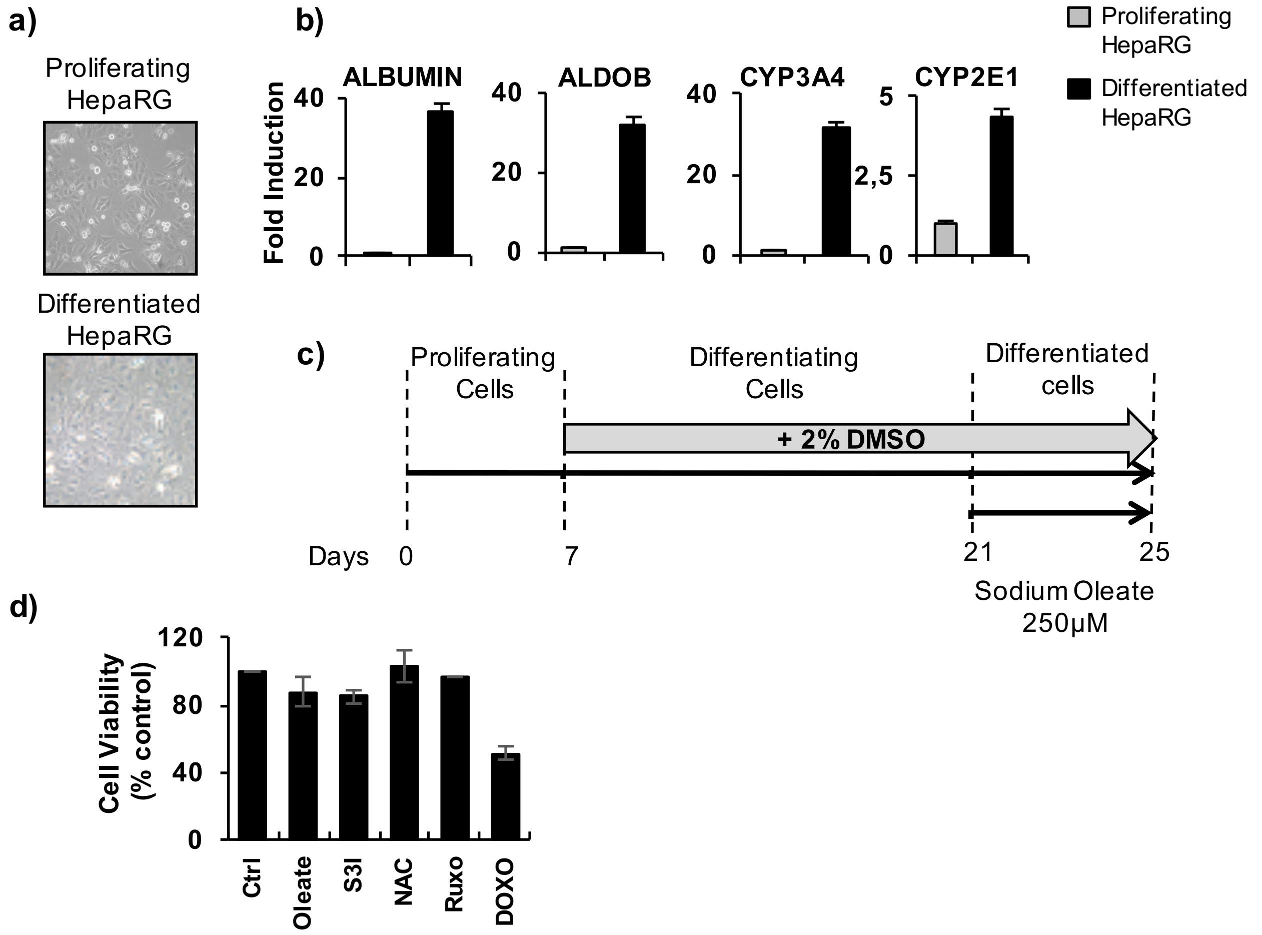

Supplementary Fig. 1. a) Picture showing proliferating human HepaRG cells (upper) and differentiated HepaRG (dHepaRG) for 15 days (lower). **b)** Total RNA were extracted from proliferating and dHepaRG cells and cDNA were analysed by qPCR using primers specific for the indicated genes. Samples were normalized to Actin. Histograms show fold induction of proliferating versus differentiated cells. **c)** Diagram showing HepaRG cell treatment protocol as described in Material and Methods. **d)** Cell viability evaluation of dHepaRG cells vehicle treated (Ctrl) or treated with sodium oleate 250 μM (5 days), S3I-201 100 μM (48 hours), N-acetylcisteina 10 mM (18 hours), Ruxolitinib 1 μM (18 hours), Doxorubucin 2 μM (18 hours) and then processed as described in the Material and Methods section for the MTT assay. Results are expressed as % relative to controls (bars indicate S.D.; p-value < 0,05 by Student T-test).

Supplementary Fig. 2

a)

Fatty Liver Card	
**Fold Change (vs control)**	
**Symbol**	**up**
ABCA1	1,89
ACSL5	1,98
APOB	2,20
CPT1A	2,27
CPT2	1,69
CYP2E1	1,79
FOXA2	2,08
IL1B	1,64
IL6	3,42
MTOR	2,25
NFKB1	1,80
PDK4	6,50
PIK3CA	2,07
PPA1	1,59
SERPINE1	2,14
	**down**
APOC3	0,47
FASN	0,49
PKLR	0,64
PNPLA3	0,52
PPARA	0,59
SCD	0,51
SLC27A5	0,64
SLC2A1	0,54
SLC2A2	0,47
SLC2A4	0,64
SREBF1	0,36

b)

IFN TLDA card	
**Fold Change (vs control)**	
**Symbol**	**up**
USP18	1,959
OAS1	4,948
ISG15	1,893
IL8	2,561
	**down**
IFIT5	0,396
MX1	0,385
GHDC	0,613
HDAC10	0,374
IFIT1	0,630
STAT2	0,641
NDUFA6	0,576
GBP1	0,561
MNT	0,533
STAT1	0,598
RFC2	0,563
PSME2	0,395
AP1G2	0,625
POLR3H	0,385
SOCS1	0,321
XAF1	0,011

c)

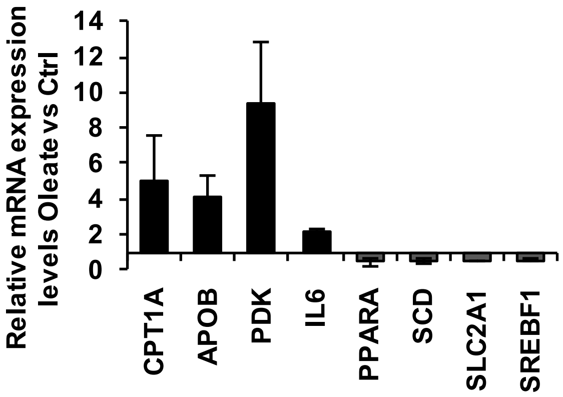

Supplementary Fig. 2. Table showing upregulated and downregulated genes from qPCR analysis of a Human Fatty Liver (a) and Interferon TLDA card (b), numbers indicate fold induction of sodium oleate treated vs control dHepaRG cells. c) Independent validation by qPCR of some of the array data presented in a/b.

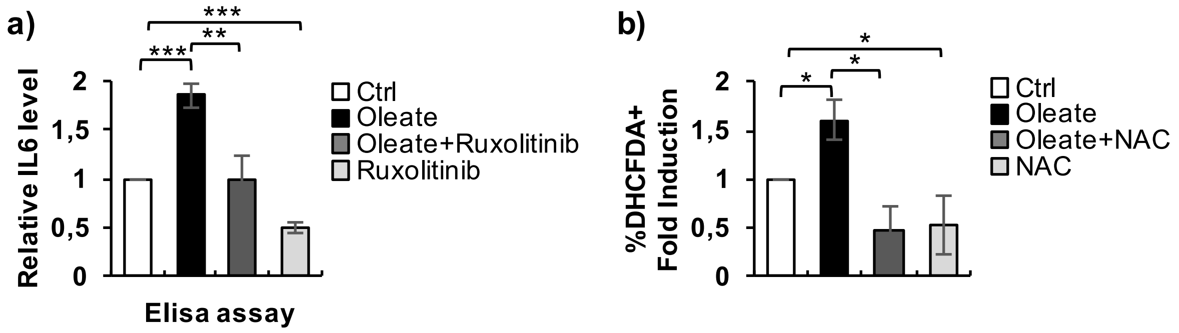

Supplementary Fig. 3. a) dHepaRG cells were treated with vehicle (Ctrl) or with sodium oleate 250 µM for 4 days, and co-treated for the subsequent 18 hours with sodium oleate 250 µM and with or with Ruxolitinib 1 µM. Supernatants were colllected to quantify secreted IL6 levels by ELISA assay. Histograms show fold induction of treated cells versus control cells. **b)** dHepaRG cells were treated with vehicle (Ctrl) or with sodium oleate 250 µM for 4 days, and co-treated for the subsequent 18 hours with sodium oleate 250 µM and with or with NAC 10mM. Cells were analyzed by citofluorimetry after DHCFDA (2',7'-dichlorodihydrofluorescein diacetate) dye staining. Histograms show % of DHCFDA positive cells as fold induction of treated versus control cells. (Bars indicate S.D.; asterisks indicate p-value).

Supplementary Fig. 4

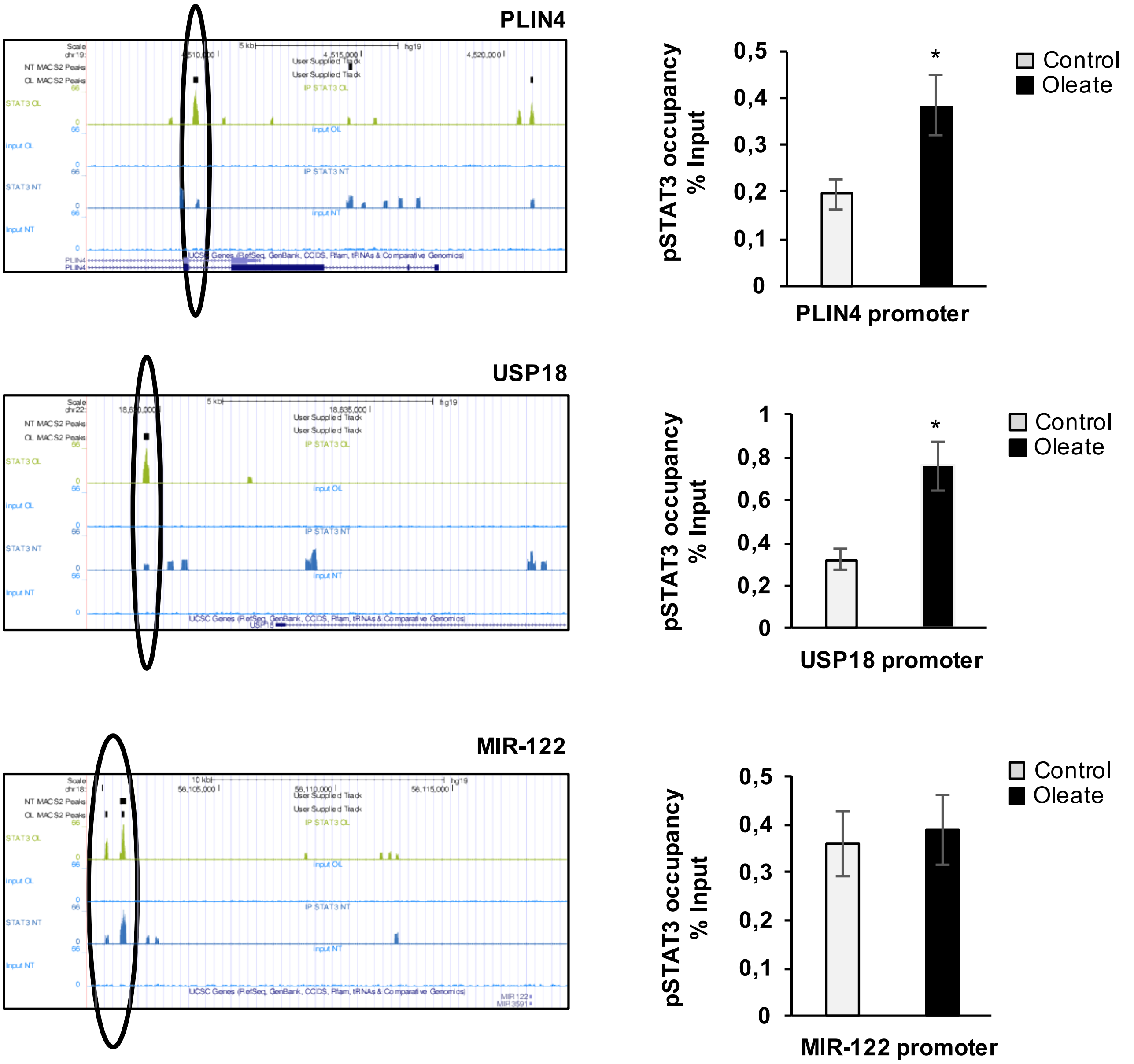

Supplementary Fig. 4. Left panels: Chip-seq profiles of pSTAT3 enrichment (black circles) on PLIN4, USP18 and miR-122 promoter of dHepaRG cells treated with sodium oleate for 4 days. **Right panels:** standard ChIp assay validation results. Cross-linked chromatin from sodium oleate or vehicle treated dHepaRG cells was immunoprecipitated with a relevant control IgG or with a pSTAT3 specific antibody and analyzed by qPCR with primers specific for PLIN4, USP18 and miR-122 promoter. ChIP results are expressed as % of Input and histograms show mean from 3 independent experiments (bars indicate S.D.; p-value $* < 0.05$ by Student T-test).

Supplementary Fig. 5

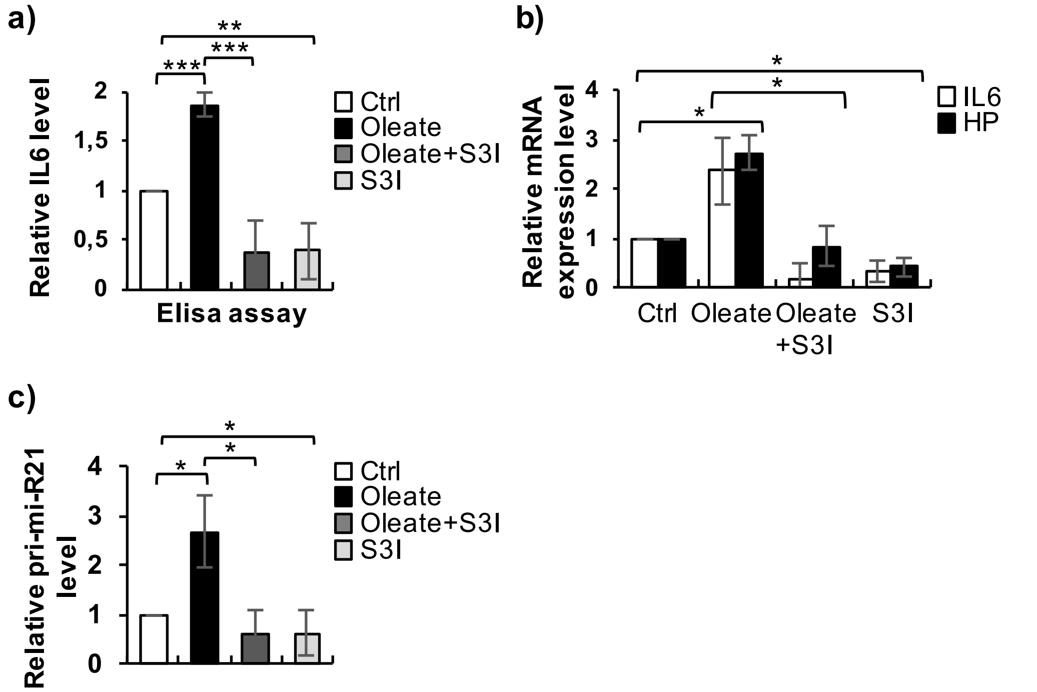

Supplementary Fig. 5. dHepaRG cells were treated with vehicle (Ctrl) or with sodium oleate 250 µM for 4 days, and co-treated for the subsequent 18 hours with sodium oleate 250 µM and with or with S3I-201 100 µM. **a)** Supernatants were colllected to quantify secreted IL6 levels by ELISA assay. **b)** cDNAs were analyzed by qPCR with primers specific for the indicated genes and normalized to Actin. **c)** Total RNA were extracted and pri-miR-21 levels were analyzed by qPCR (TaqMan MicroRNA Assay, Applied Biosystems) and normalized to GAPDH. Histograms show fold induction of treated cells versus control cells; bars indicate S.D.; asterisks indicate p-value.

Supplementary Fig. 6

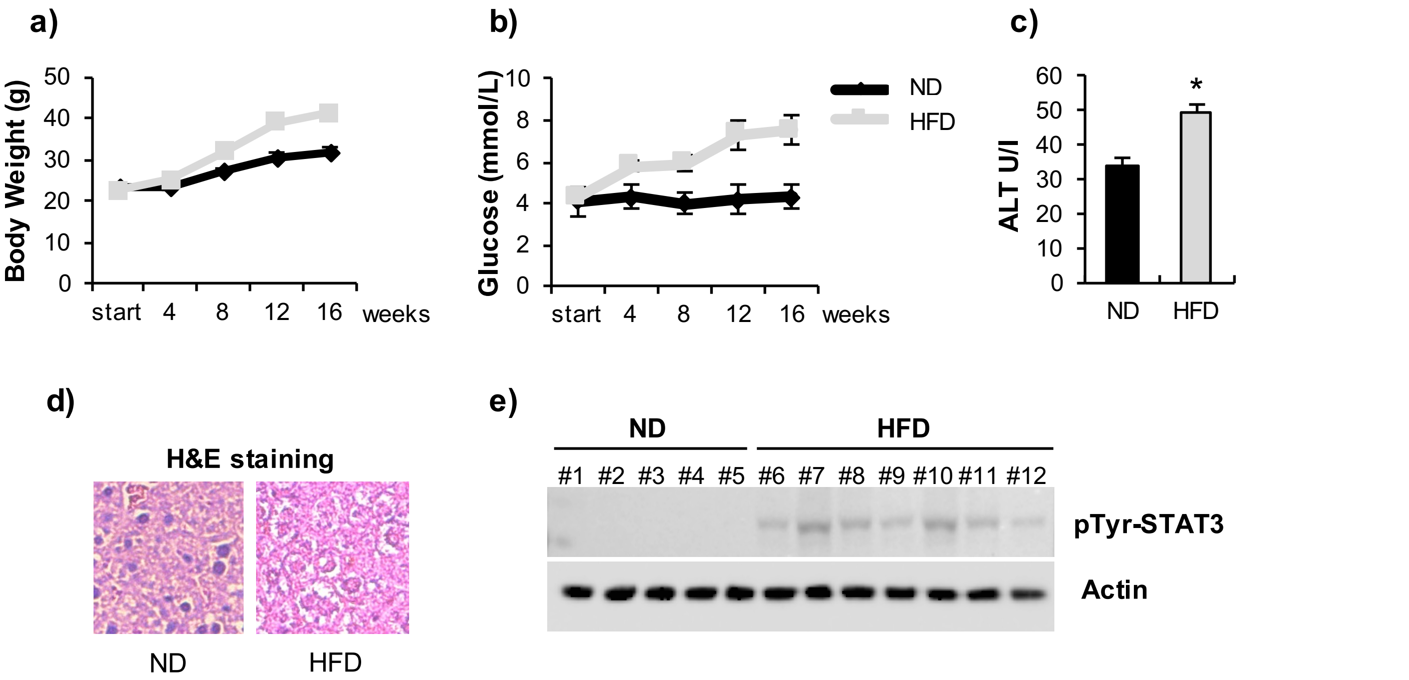

Supplementary Fig. 6. C57/BL6 mice were fed normal diet (ND) or High-Fat diet (HFD) (59% fat, 15% protein, 26% carbohydrate) for 16 weeks (7 mice per group). **a/b)** Animals' body weight and Glucose levels (mmol/L) was measured weekly to monitor HFD effects vs ND until sacrifice at 16 weeks. **c)** Measurement of ALT (alanine aminotransferase) liver enzyme activity from 16 weeks HFD and ND mice. **d)** Hematoxylin/Eosin staining from mice liver tissues. **e)** Protein extracts from ND and HFD mice liver tissue were analysed by immunoblot with pTyr-STAT3 antibody and normalized to Actin protein levels.

Supplementary Fig. 7

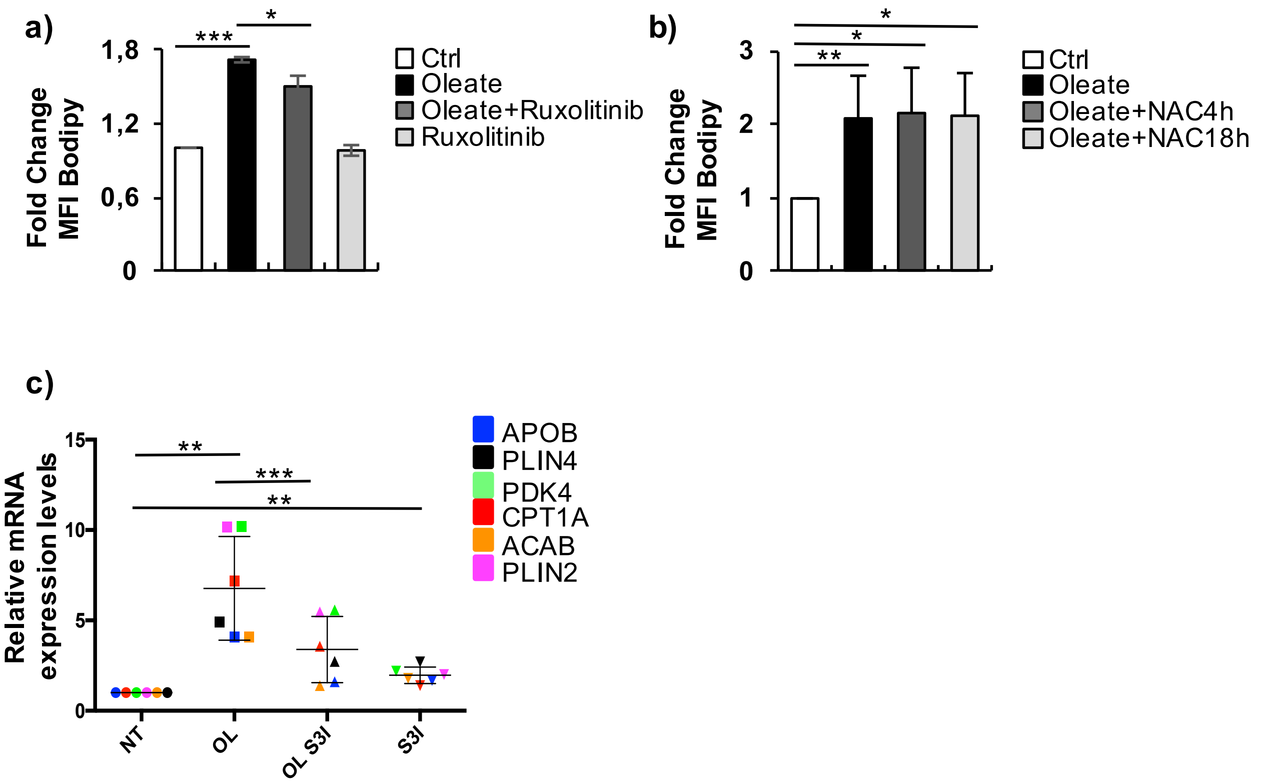

Supplementary Fig. 7. a) FACS analysis using the lipophilic dye Bodipy to quantify neutral lipid production. dHepaRG cells were treated with DMSO (control) or with sodium oleate for 4 days, and for the subsequent 18 hours co-treated with sodium oleate plus Ruxolitinib 1 µM. **b)** FACS analysis as in a). dHepaRG cells were treated with DMSO (control) or with sodium oleate for 4 days, and for the subsequent 4 or 18 hours co-treated with sodium oleate plus NAC 10mM. Histograms show fold induction of Bodipy MFI (Mean Fluorescent Intensity). **c)** dHepaRG cells were treated with sodium oleate 250 µM for 4 days or vehicle treated (control) and co-treated for the subsequent 48 hours with sodium oleate 250 µM plus S3I-201 10 µM or S3I-201 alone. cDNAs were analyzed by qPCR with primers specific for the indicated genes and normalized to GAPDH, results are expressed as fold induction of treated cells versus control cells (the horizontal line indicates the mean value and whiskersbars indicate S.D.; asterisks indicate p-values).

Supplementary Fig. 8

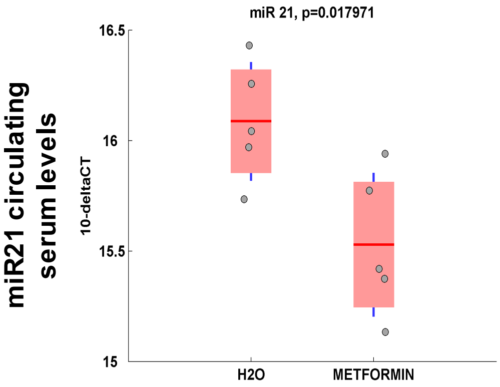

Supplemenatary Fig. 8. 5 weeks aged C57/BL6 mice were daily treated with either water or metformin (50 mpk) for 72 weeks. Boxplot represents q-PCR quantification of circulating miR-21 expression in blood from mice at the end of treatment (77 weeks). The C. Elegans miR-39 was used as control to standardize miRNAs expression. Results are expressed as 10^{-DCt}.

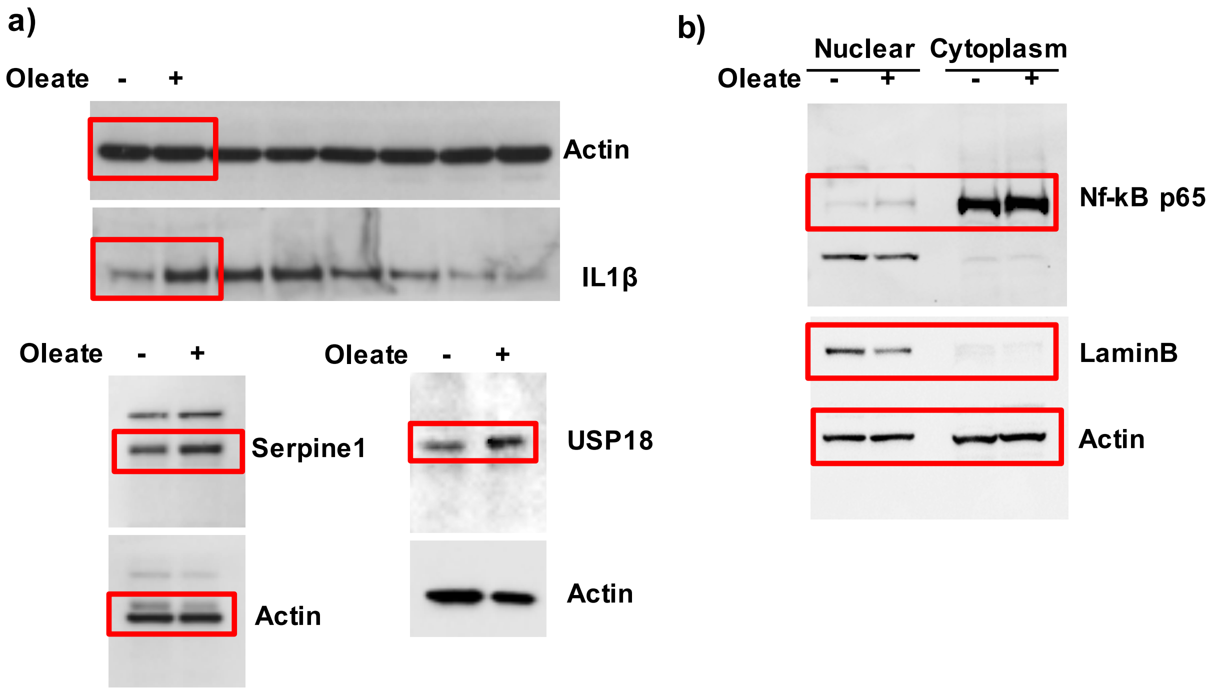

Supplementary Fig. 9. Full-lenght blots for Figure 2. **a)** Uncropped blot for Fig. 2b. **b)** Uncropped blot for Figure 2d.

Supplementary Fig. 10

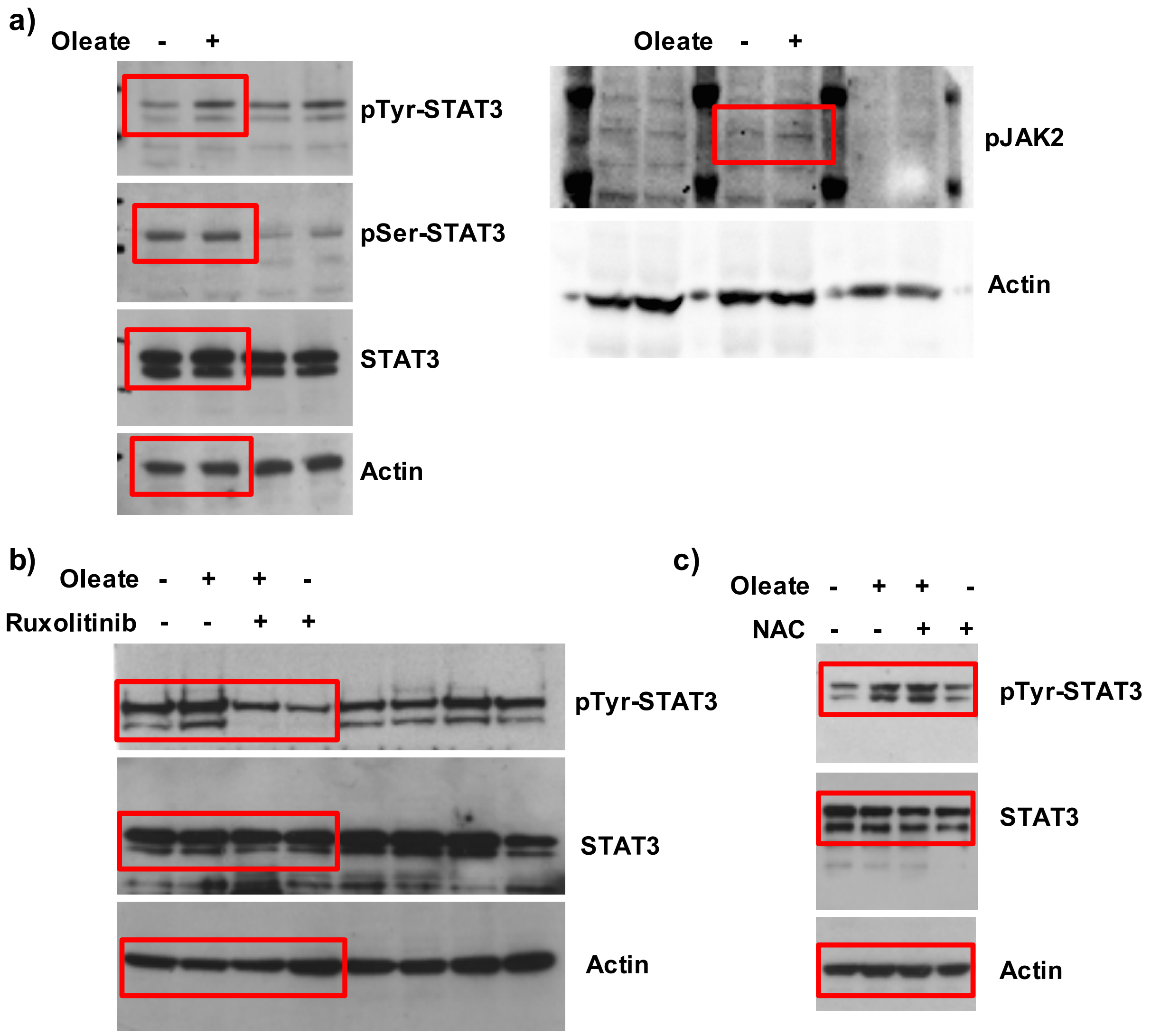

Supplementary Fig. 10. Full-lenght blots for Figure 3. **a)** Uncropped blot for Fig. 3a. **b)** Uncropped blot for Figure 3c. **c)** Uncropped blot for Figure 3d.

SUPPLEMENTARY TABLES

Supplementary Table 1

TLDA card	Fatty Liver card
ADAR, AP1G2, ATF5, BAG3, BAX, BCR, BST2, CAV1, DAD1, DDX58, DUSP1, EEF1G, EIF2AK2, GBP1, GBP2, GGA1, GHDC, GNAZ, HDAC10, HERC5, HIF1A, HTATIP2, IFI16, IFI27, IFI35, IFI44, IFIH1, IFIT1, IFIT2, IFIT3, IFIT5, IFITM2, IL17RA, IL8, IRF1, IRF9, ISG15, LAP3, LGALS3BP, MET, MNT, MRPS28, MX1, MYD88, NDUFA6, OAS1, OAS3, PIAS4, PIK3AP1, PLEC, PLSCR1, PNN1, POLR3H, PSME1, PSME2, RBX1, RFC2, RPLP2, SAMM50, SF3A1, SGSM3, SLC25A1, SMARCB1, SOCS1, SOCS3, SP110, ST13, STAT1, STAT2, STXBP5, TNFSF10, TRMU, UBE2L6, USP18, WARS, XAF1	ABCA1, ACACA, ACADL, ACLY, ACOX1, ACSL5, ACSM3, ADIPOR1, ADIPOR2, AKT1, APOA1, APOB, APOC3, APOE, ATP5C1, CASP3, CD36, CEBPB, CNBP, CPT1A, CPT2, CYP2E1, CYP7A1, DGAT2, FABP1, FABP3, FABP5, FAS, FASN, FOXA2, FOXO1, G6PC, G6PD, GCK, GK, GSK3B, HMGCR, HNF4A, IFNG, IGF1, IGFBP1, IL10, IL1B, IL6, INSR, IRS1, LDLR, LEPR, LPL, MAPK1, MAPK8, MLXIPL, MTOR, NDUFB6, NFKB1, NR1H2, NR1H3, NR1H4, PCK2, PDK4, PIK3CA, PIK3R1, PKLR, PNPLA3, PPA1, PPARA, PPARG, PPARGC1A, PRKAA1, PTPN1, RBP4, RXRA, SCD, SERPINE1, SLC27A5, SLC2A1, SLC2A2, SLC2A4, SOCS3, SREBF1, SREBF2, STAT3, TNF, XBP1

Supplementary Table 1. Table showing complete list of genes included in the TLDA and Fatty Liver cards.

Supplementary Table 2

	Control	*Oleate*	*Oleate + S3I*	*S3I*
**Total number of cells**	**222**	**164**	**168**	**81**
Droplet count (all droplets in all cells)	588	9884	5413	59
Mean number of droplets per cell	3	60	32	1
Standard error	2	6	6	0
Mean droplet area (all droplets in all cells, μm^2)	0,145	0,131	0,099	0,035
Standard error (μm^2)	0,022	0,005	0,004	0,005
Mean of total droplet area per cell (μm^2)	0,38	7,88	3,2	0,03
Standard error (μm^2)	0,24	1,23	0,80	0,02
Mean of percentage droplet area per cell	0,05	0,76	0,32	0
Standard error	0,03	0,11	0,05	0,00

Supplementary Table 2. Table showing CARS analysis data from dHepaRG cells treated as indicated.
